# Supplementary material for: Deletion of wfs1 Impairs Oligodendrocyte Precursor Cells Dorsal Distribution and Myelination Through the wfs1-hmgcs1 Axis in Zebrafish
Source: Biology (Basel). 2026 Aug 16;15(16):1402. doi: 10.3390/biology15161402 (PMC13510151; doi:10.3390/biology15161402)
Supplement: Supplementary file 1 [file biology-15-01402-s001.zip › File S1-Transcriptome sequencing data and differentially expressed genes (DEGs) in wfs1-- zebrafish at 5 dpf/2.KEGG/wfs1KOvsWT/DOWN/src/dre04218.html]

dre04218


- dre:557551

- Down regulated genes

557551(-1.32931971737724)
- dre:572042

- Down regulated genes

572042(-1.37390437938079)

- dre:100000252

- Down regulated genes

100000252\_1(-1.7718817168434)

- dre:431763

- Down regulated genes

431763\_1(-4.20440659354098)

- dre:393176

- Down regulated genes

393176\_1(-2.88755255820479)

- dre:100037341

- Down regulated genes

100037341(-1.07503837943821)

- dre:565241

- Down regulated genes

565241\_2(-6.09874532631292)

- dre:100002946

- Down regulated genes

100002946(-1.40391573648291)

- dre:100000252

- Down regulated genes

100000252\_1(-1.7718817168434)

Close
